# Book Reviews

**Published:** 1902-09

**Authors:** 


Practical Dietetics. With Special Reference to Diet in Disease. By W. Gilman Thompson, M. D., Professor of Medicine in Cornell University Medical College, New York City; Visiting Physician to the Presbyterian and Bellevue Hospitals. Octavo, pp. 851. Second edition, enlarged and thoroughly revised. New York: D. Appleton & Company. 1902. [Price, $5.00.]

Food studies comprise an important topic in the scientific series of experimental investigations of the period, and a knowledge of food values in dietetics is growing to be a necessity. This is as true whether in the positive sense of what should be eaten in health and disease, or in the negative meaning of what should be avoided in either condition.

To state the case in another way the economic values of dietaries as applied to the several wasting diseases, is of even more importance from a clinical viewpoint than the therapeutic values of the several drugs that may have more or less clinical importance.

When the first edition of this work appeared it was hailed as the first systematic treatise on the subject that had reached the textbook dignity, and it was adopted as such by the colleges to a considerable extent. This treatise has taken the lead, therefore, in creating an interest in dietetics, far beyond the former condition of the science, and a scientific application is being made of the principles taught by Thompson, in almost every department of medicine.

Before the publication of this textbook the literature of the subject was scattered throughout treatises upon internal medicine and in monographs, and the science of feeding patients properly was in very crude shape. Now, thanks to this author, we have reached a more systematic basis and can prescribe food with nearly as much precision as we can medicine.

Professor Thompson is a teacher of experience and a master of his work. In this edition he has presented much new material and has revised and rewritten—one or both—much of the old, so that it represents the condition of the science today. It is a work that should be in the hands of every physician no matter what his specialty or "system" of practice,

---

Transactions of the American Association of Obstetricians and Gynecologists. Vol. XIV. For the Year 1901. Edited by William Warren Potter, M. D. Philadelphia: William J. Dornan, Printer. 1902. Copyrighted.

The annual volumes of the association are replete with interest and evince a high scientific *esprit de corps* on the part of its members. This handsomely printed and bound standard octavo book of over 400 pages contains the proceedings of the association, including the papers read and discussions thereon, at its fourteenth annual meeting at Cleveland, September 17, 18 and 19, 1901. Meeting, as it did, under the shadow of the great national affliction that had then just befallen this country, the funeral of President McKinley being held while the association was in session, it is almost remarkable that the proceedings were completed. Nevertheless, even in the face of such dire calamity, this volume is one of the best issued by the organisation. From contents page to index, from president's address to memorials, it is full of instructive interest.

Such a meeting by such a body of trained medical men furnishes in reality a post-graduate course of instruction to the audience, and we are pleased to note that the list of visitors in attendance at this meeting was a long one. It should be so. The medical profession in the vicinity of these meetings should attend in full force, for in no other way in the same length of time can as much valuable information be obtained by the general practitioner or by him who would become a specialist, or by one who now maybe is a semi-specialist.

The association made its annual dinner a memorial occasion at which fitting tributes were paid to the life and character of the dead president, one of which was a most feeling address by one of President McKinley's very intimate friends. While this may seem somewhat bizarre when related, yet as witnessed it really was a most appropriate ceremonial entirely befitting the solemn ceremonies—occasioned by the great tragedy—beginning in Buffalo and ending at Canton during the week of this meeting.

The next meeting to be held at Washington during this month, as noticed in the JOURNAL for August, promises to be one of unusual interest.

Quain's Dictionary of Medicine. By various writers. Edited by H. Montague Murray, M. D., F. R. C. P., Joint Lecturer on Medicine, Charing Cross Medical School and Physician to Out-patients, Charing Cross Hospital; Senior Physician to the Victoria Hospital for Children, Chelsea, and to the Foundling Hospital. Assisted by John Harold, M. B., B. Ch., B. A. O., Physician to St. John's and St Elizabeth's Hospitals, and Demonstrator of Medicine at Charing Cross Medical School; and W. Cecil Bosanquet, M. A., M. D., M. R. C. P., Physician to Out-patients, Victoria Hospital for Children, Chelsea, and Pathologist to Charing Cross Hospital; Late Fellow of New College, Oxford. Third edition, largely rewritten and revised throughout. With 14 colored plates and numerous other illustrations. New York: D. Appleton & Co. 1902. [Price, half-morocco. $10.00.]

During the past twenty years many reference books have been created but it is quite safe to say that none have excelled Quain's in practical value. Some, to be sure, have been more comprehensive extending through a series of tomes, while others have been issued in periodical form. But no single volume work equals this in comprehensiveness, scientific accuracy, minuteness of detail or clearness of expression.

From the first it became a favorite with the profession and its general scheme as originally laid out by Sir Richard Quain has been steadily maintained. One of its strong points is the development of diagnosis and treatment which will be found running through every article to which those topics are germane.

In this edition many articles have been rewritten, some have been omitted, while others have been revised, the effect of which is to present a thoroughly modern volume which may serve to adorn any physician's library. The list of contributors is a long and able one embracing the names of some of the most eminent writers known in the literature of medicine. The editor of the present edition has certainly distinguished himself.

THE SURGERY OF THE RECTUM. By CHARLES B. KELSEY. A. M., M D., Late Professor of Pelvic and Abdominal Surgery at the New York Post-Graduate Hospital, and Professor of Rectal Surgery at the University of Vermont. Octavo, pp. 411. Sixth edition. Illustrated by 215 engravings. New York: William Wood & Co. 1902. [Price, 3.00]

This work is no stranger to the profession, it having stood the test of five previous editions in the period of about twenty years. The first edition was one of a series of monographs published by William Wood & Company and appeared in 1882.

This edition is largely a rewritten issue as the changes necessary to bring it forward to the present time were too numerous to prevent the author to remain content with a mere revision.

The old general style and characteristics are largely maintained, but it becomes now a modern treatise in all essentials.

The illustrations are numerous. There is considerable gynecology dealt with in it for a work with its title, but that does not detract from its value—it even enhances it for many persons.

---

Seventeenth Annual Report of the State Board of Health and Vital Statistics of the Commonwealth of Pennsylvania. Vols. I. and II. Transmitted to the Governor, November 30, 1900. Benjamin Lee, M. D., Secretary. Wm. Stanley Ray, State Printer of Pennsylvania. 1901.

The well organised and equally well administered Pennsylvania State Board of Health issues each year an elaborate and scientific report of its work. It embraces reports from the boroughs and cities throughout the state, so that in the two volumes can be found the true sanitary status of every county and populous region within the commonwealth.

Almost every public health question is discussed in these annual reports, and school sanitation, a topic of vast moment, is given special prominence. The secretary of the Pennsylvania board is a sanitarian of superior attainment as well as a secretary and executive officer of vast experience, hence the great value of his annual volume.

---

Johnson's First Aid Manual. Suggestions for Prompt Aid to the Injured in Accidents and Emergencies. Edited by Fred B. Kilmer. Illustrated Pp. 113. New Brunswick, N. J.: Johnson & Johnson. 1901. [Cloth, 50 cents.]

This little brochure is full of practical thought that will serve as a reminder to the medical men who are liable to meet with occasional accidents and to render the surgical care resultant therefrom. It is designed to accompany Johnson's accident case, and deals principally with the surgical appliances made by the celebrated house of Johnson & Johnson. The manual is full of excellent illustrations, largely drawn from life or real conditions, which show with distinctness many of the dressings in situ.

